# Association between blood lead levels and markers of calcium homeostasis: a systematic review and meta-analysis

**DOI:** 10.1038/s41598-022-05976-4

**Published:** 2022-02-03

**Authors:** Kuldip Upadhyay, Ankit Viramgami, Bhavani Shankara Bagepally, Rakesh Balachandar

**Affiliations:** 1 ICMR – National Institute of Occupational Health, Ahmedabad, India; 2 ICMR – National Institute of Epidemiology, Chennai, India

**Keywords:** Environmental sciences, Diseases, Endocrinology, Health care, Health occupations, Pathogenesis, Risk factors

## Abstract

Chronic Pb exposure associated systemic illness are partly posited to involve calcium homeostasis. Present systematic review aims to comprehensively evaluate the association between chronic lead exposure and markers of calcium homeostasis. Observational studies documenting the changes in calcium homeostasis markers (i.e. serum calcium, parathyroid hormone, vitamin D & calcitonin) between occupationally Pb exposed group and control group were systematically searched from pubmed-Medline, Scopus, and Embase digital databases since inception to September 24, 2021. The protocol was earlier registered at PROSPERO (ID: CRD42020199503) and executed adhering to PRISMA 2020 guidelines. Mean differences of calcium homeostasis markers between the groups were analysed using random-effects model. Conventional *I*^2^ statistics was employed to assess heterogeneity, while the risk for various biases were assessed using Newcastle Ottawa Scale. Sub-group, sensitivity and meta-regression analyses were performed where data permitted. Eleven studies including 837 Pb exposed and 739 controls were part of the present study. Pb exposed group exhibited higher mean blood lead level [i.e. 36.13 (with 95% CI 25.88–46.38) µg/dl] significantly lower serum calcium (i.e. − 0.72 mg/dl with 95% CI − 0.36 to − 1.07) and trend of higher parathyroid levels and lower vitamin D levels than controls. Heterogeneity was high (*I*^2^ > 90%) among the studies. Considering the cardinal role of calcium in multiple biological functions, present observations emphasis the need for periodic evaluation of calcium levels and its markers among those with known cumulative Pb exposure.

## Introduction

Lead (Pb), a common hazardous heavy metal bioaccumulates in various tissues on chronic exposure, causing serious systemic illnesses. The pathophysiology posited in some Pb-induced health hazards involves direct and indirect interference with calcium metabolism and homeostasis. Despite very few studies verify the causal role of perturbations on calcium homeostasis/metabolism; collective evidence support the Pb-calcium interactions^1^. The availability of calcium in serum for various biological functions are tightly regulated by parathyroid hormone (parathormone), calcitonin and vitamin. Therefore, parathormone, calcitonin, vitamin D, and serum calcium (itself) constitute calcium homeostasis markers^2^.

One of the mechanism posited in Pb altering calcium homeostasis include inhibition of enzymes responsible for vitamin D activation (i.e., 1 α-hydroxylase), thereby reducing serum calcium^3,4^. Given growing reports of Pb among the general population and inconclusive evidence from individual primary studies regarding the association of blood lead levels (BLL) on calcium homeostasis markers (parathyroid and vitamin D)^5–9^, there is need to investigate current evidences of association (if any) between the duo.

Individuals occupationally exposed to Pb and its derivatives commonly exhibit high BLL. Hence, the current study aimed at systematically reviewing the studies comparing calcium homeostasis markers between individuals occupationally exposed to Pb as against those without obvious Pb exposure. In addition the study aimed to explore the association between BLL and bone resorption/turnover markers (urinary pyridinoline, urinary deoxypyridinoline, osteocalcin, osteopontin).

## Methods

Current systematic review aimed to investigate the association between calcium homeostasis markers and occupational Pb exposure. We synthesized results of the studies conducted among the adult population (P), occupationally exposed to Lead (Exposure/intervention) compared to obviously unexposed to Lead (comparator), reporting the changes in outcomes parameters viz. calcium, vitamin D, calcitonin and parathyroid. The study employed PRISMA (“Preferred Reporting Items of Systematic reviews and Meta-Analysis”) during execution of the study^10^ and the study proposal was registered at PROSPERO (ID: CRD42020199503). Observational or interventional studies of either cross-sectional or longitudinal design, evaluating calcium homeostasis markers among occupationally Pb exposed participants as against those without obvious Pb exposure were systematically searched using search terms, from Embase, Pubmed-Medline and Scopus digital repositories. The search terms were developed using PICOS approach (i.e., Participants, Intervention (i.e. occupational Pb exposure), Controls (no obviously Pb exposure), Outcome (calcium homeostasis markers), and Study design). Relevant studies were identified using precision and sensitivity maximizing strategies. Detailed search strategies and the search words/terms used in current study is described in the appendix. The repositories were initially searched on September 24, 2020 and later updated on March 17, 2021. A lateral search was performed for potential studies using the bibliography of included studies.

Primary literature reporting the differences in calcium homeostasis markers in otherwise healthy individuals occupationally exposed to Pb, as compared to those without a history of occupational Pb exposure, were considered. Calcium homeostasis markers viz. serum calcium, vitamin D, calcitonin, and parathyroid were primarily aimed, bone resportion/turnover markers were additionally considered during the search. Preclinical studies (animal/cell line), studies reporting acute Pb poisoning, involving individuals with congenital/acquired disorders of calcium metabolism or other endocrine disturbances, review articles, commentaries, case studies, letters to the editors, editorials, and methodology articles were removed from the review.

### Screening and reviewing of studies

All records obtained from various digital repositories were pooled and uploaded at cloud based “Rayyan intelligent systematic review” for independent screening of titles and abstract by all authors. Duplicate records were identified by the “Rayyan intelligent systematic review” and made available for manual confirmation to individual authors (RB, KU and AV)^11^. The title and abstract of all records were initially screened independently for their potential inclusion. Full text of these identified records were independently reviwed (RB, KU and AV). Records meeting the inclusion–exclusion criteria were indentified for data extraction ensuring no duplication. All conflicts during the independent review were resolved by mutual consensus.

### Data extraction, analysis, and management

All essential details from the selected primary articles necessary to execute the proposed study were extracted using Google sheet based data extraction sheet (DES). The DES included details of participant (age, gender, and duration of employment), exposure/occupation/workplace (nature of workplace, location, additional heavy metal(s) exposure), and outcome measurements, in addition to the details of the article (title, author(s), journal, published year, and email of the author(s)) whenever available. The protocol for studies meeting inclusion criteria with either incomplete/no data involved contacting the corresponding/primary authors for sharing the relevant data.

Central tendency values (median/mean) and data dispersion (Standard error (SE)/standard deviation (SD)/95% confidence interval (CI)/Interquartile range) for the outcome parameters were independently extracted from all included studies (AV & RB). Webplot application^12^ was used for extracting data when the results were graphically reported^13^. All recorded data were confirmed for consistency before executing further analysis. In situations where the outcome parameters were reported in unconventional/non-standard units, standard conversion factors were used for converting them to standard units^3,6^. The calcium, lead, vitamin D and parathyroid levels were converted to the SI units, i.e. mg/dl, µg/dl, ng/dl and pg/dl respectively.

When central tendency values (i.e. mode, median) & the data dispersion (e.g., 95% confidence interval, interquartile range, standard error of mean) were available^14^ alternate to mean & SD, standard conversions were adopted for uniform reporting^15–17^. Lastly, in situations where the primary study reported more than one exposed group^7^, grand mean and SD were calculated^18^, Pooled mean difference (95% CI) between the occupationally Pb exposed workers and the control group was estimated using the generic inverse variance method by pooling the mean and SD from individual studies. On anticipating high heterogeneity random-effects model was employed during meta-analysis^19^.

### Sensitivity, heterogeneity, subgroup analyses and Risk of Bias (RoB) assessment

Cochran-Q test, I-squared (*I*^*2*^) statistics and visual inspection of the forest plots were employed to assess the heterogeneity among included studies. The *I*^*2*^ > 25% or Cochrane-Q < 0.1 were regarded as evidence of heterogeneity among the included studies^20^. Further, the sources of heterogeneity were explored by fitting the co-variables (mean age, mean duration of exposure, and percentage of males in the study, whichever was available) individually in the meta-regression model when more than ten primary studies were available. A particular variable exhibiting a 50% reduction in *I*^2^ was regarded as a potential source of heterogeneity.

Egger's test (*P* < 0.05) of the effect measures and funnel plot (asymmetry) were used for assessing the publication bias^21,22^. Contour-enhanced funnel plot was used to explore the source of asymmetry on encountering asymmetric funnel plot. Lastly, subgroup and sensitivity analyses were undertaken to evaluate the influences of additional exposure to other heavy metals (yes *vs.* no/not available) on the outcome variables. However, the sub-group analysis was executed on availability of sufficient (i.e. ≥ 2) studies. The results of particular subgroup analysis was interpreted considering the change (magnitude & direction) in mean differences and heterogeneity (i.e. Cochran-Q test, *I*^*2*^ statistics)^23^.

Microsoft Excel sheet (version 2016) was used for data recording and Stata (16th version) was used for all analysis^24^. Statistical significance was considered at *P* < 0.1 for subgroup analyses, while for the rest of analyses, *P* < 0.05 was regarded as statistically significant.

### Risk of Bias (RoB) assessment

Newcastle Ottawa Scale (NOS) was employed independently by the authors for assessing ROB in the included studies^25^. Briefly, the NOS assessed the risk of bias during comparing cases and controls, participant selection and exposure assessment in the primary studies. Based on agreement with the definition of cases and controls in the individual studies, their representativeness and comparabality, and relative occupational Pb exposure, individual studies were rated. Conflict during RoB assessment was resolved by mutual consensus among the authors.

### Certainty of evidence assessment

The level of certainty for each of the outcome parameters investigated in the present study were evaluated as per Grading of Recommendations Assessment, Development and Evaluation (GRADE) guidelines^26^. The tool aids in summarising the quality of evidence in the structured process, facilitating the interpretation of results. GRADE assessment broadly involves 4 categories including “High”, “Moderate”, “Low” and “Very Low” based on risk of biases including the publication bias, inaccuracy and inconsistency in the outcome variables. Conventionally the quality of evidence for observational studies are very low–low, however in exceptions where the magnitude of effect is large the certainty of evidence may improve^26^.

## Results

**Figure 1:**
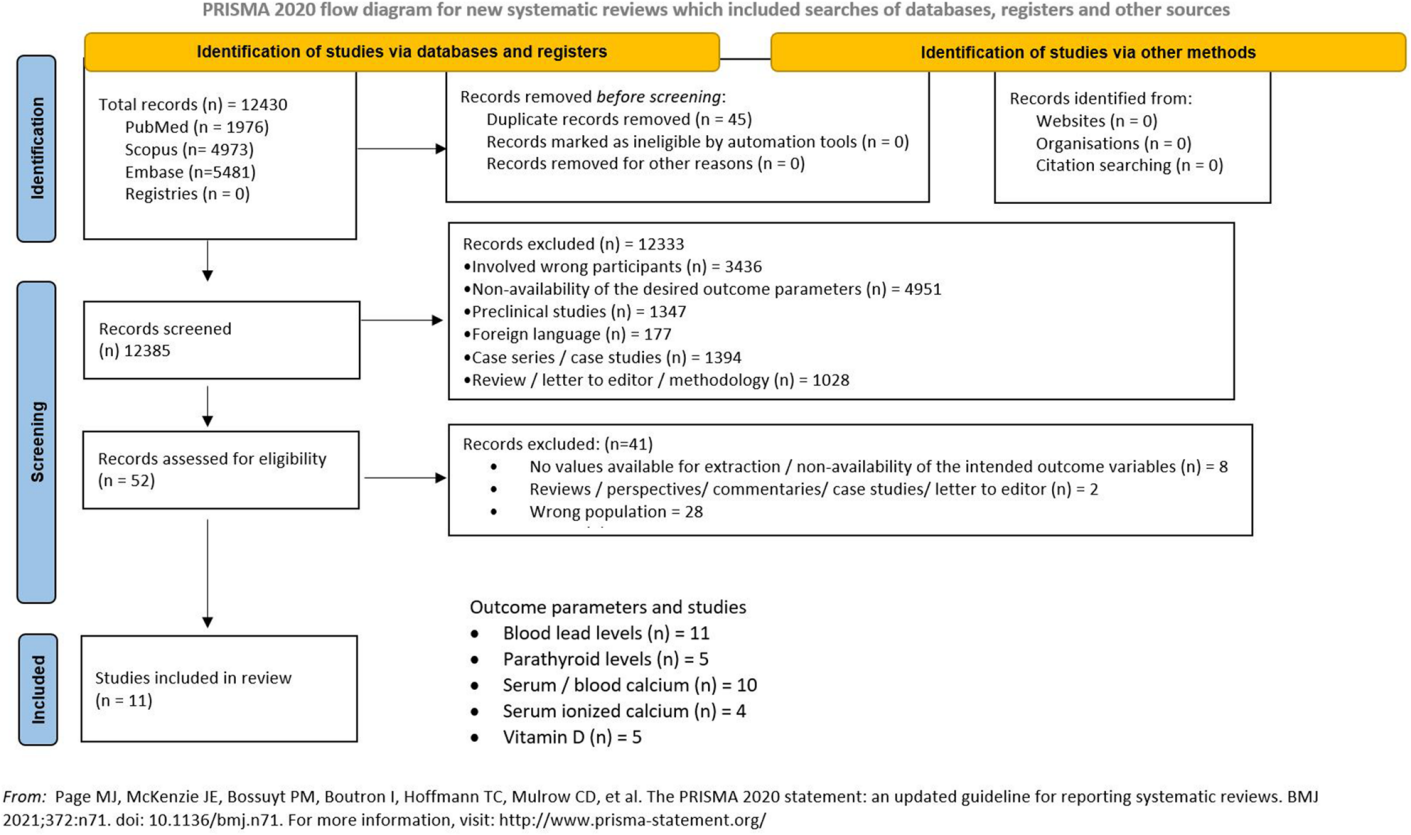
PRISMA flow chart. Legends: PRISMA flow chart illustrating the number of records included and excluded at various screening and revieweing steps, leading to final list of records for data extraction and meta-analysis.

The electronic search retrieved 12,430 records. A total of 52 records (Supplementary table 4) were identified for full text review after excluding 45 duplicate records and 12,333 records during screening of abstract & titles. Title and abstract screening revealed, 3436 records with participants not exposed to occupational Pb exposure/comparative group, 1347 records involving animals/invitro study designs, 1394 records with brief reports from various case series and studies, 1028 records from reviews, methodologies and others, 177 records reported in languages other than English and 4951 records reporting parameters (e.g. genetic studies, bone Pb levels, etc.) other than the parameters of interest to the present study. While no records related to registering the study protocols relevant to current study were available. Full-text scrutiny resulted in eleven studies for the final quantitative data synthesis. Total number of studies excluded during each screening/reviewing phases and the reasons are available in PRISMA flowchart (Fig. 1). All studies meeting the inclusion criteria reported necessary data for current study.

### Description of studies

Individual study details including study site (country), proportion of samples in exposed and control and their mean age, duration of occupational exposure, type of industry (responsible for occupational Pb exposure), additional exposure to other heavy metals, mean (SD) calcium levels in the participants and outcome parameters are described in Table 1. Mean outcome variables of the control group from majority studies were within recommended normal values (i.e., Serum calcium levels: 8.2–10.2 mg/dl, ionized Calcium levels: 4.60–5.08 mg/dl, serum PTH: 10–65 pg/ml and vitamin D: 25–80 ng/ml)^27^ suggesting the control group as apparently normal in terms of these outcome parameters^27^. However, the mean Vitamin D of the control group from Dongre et al. 2013 was lower than the recommended values^7^. None of the studies reported calcitonin levels, while an individual study reported bone turnover/ resorption markers^5^. Briefly, all included studies were cross sectional, observational, comparative design involving 100% male participants, except Batra et al.^4^, where 11.3% were female participants. The sources of occupational Pb exposure among the exposed group vaired across Pb battery and its allied industries, Pb smelting process, e-waste recycling process, welding, and paint industries. The duration of occupational Pb exposure was not available for majority of the studies, while few reported the exposure duration ranging from 5 to 34 years. The studies were restricted to Asian (India, China, and Bangladesh), European (Croatia, Austria, Turkey, and Israel), and African (Nigeria) countries, while no studies from Australia or American (both Latin and North) countries were available.

**Table 1:** Description of the studies.

Study	Country	Outcome parameters reported	Sample Size (n) [Exposed: controls]	Age in years (mean ± SD) [Exposed Vs Controls]	Serum Ca levels in mg/dl (mean ± SD) [Exposed Vs Controls]	Duration of Pb exposure (in years) [mean ± SD]	Exposure to other heavy metals	Industry type of exposed
Akbal et al.^5^	Turkey	PTH, Calcitriol, UDP* HYP* and OC	30 : 32	39.4 ± 8.7 *Vs* 37.9 ± 8.2	NA	NA	NA	Lead batteries,
Anetor et al.^3^	Nigeria	Ca*	86 : 51	NA	8.9 ± 0.76 *Vs* 9.26 ± 0.56	NA	Copper	Lead factory
Anetor et al.^6^	Nigeria	Ca	47 : 25	33 ± 8.2 *Vs.* 36 ± 2.5	9.1 ± 0.36 *Vs* 9.7 ± 0.28	NA	Magnesium	Welding (n = 9), Printing (n = 10), Paint (n = 14) and Battery (n = 14)
Batra et al.^4^	India	Ca*, PTH*, Vit-D*	80 : 80	30.9 ± 5.75 *Vs.* 30.2 ± 6.01	8.35 ± 0.42 *Vs* 9.33 ± 0.47	8.57 ± 3.81	NA	Lead battery
Dongre et al.^7^	India	Ca, PTH, Vit-D	90 : 30	Range: 20 to 45	8.3 ± 0..7 *Vs* 9.99 ± 0.43	30 each with 1–5, 6–10 and > 10 years	NA	Lead battery
Estela Kristal Boneh et al.^8^	Israel	Ca, PTH, Vit-D, Calcitriol*	56 : 90	43.4 ± 11.2 *Vs.* 41.5 ± 9.3	9.7 ± 0.5 *Vs* 9.9 ± 0.4	5.3 ± 4.0	Magnesium	Lead battery and recycling
Himani et al.^28^	India	Ca*, Vit-D*	100 : 100	32.6 ± 10.3 *Vs.* 34.7 ± 7.9	8.8 ± 0.5 *Vs* 9.1 ± 0.8	14.8 ± 9.5	NA	Lead battery
Mazumder et al.^29^	Bangladesh	Ca*, PTH*, Vit-D*	47 : 42	NA	7.7 ± 2.6 *Vs* 9.8 ± 1.8	NA	NA	Jewellery
Osterode et al.^9^	Austria	Ca	12 : 12	42.8 ± 5.0 *Vs.* 40.1 ± 5.0	9.37 ± 0.35 *Vs* 9.58 ± 0.52	NA	NA	Pb smelting
Pizent et al.^14^	Croatia	Ca*	143 : 156	34 *Vs.* 35	9.98 ± 12.47 *Vs* 9.46 ± 8.94	8 (range 2–34)	Cadmium	Lead battery
Wang et al.^13^	China	Ca*	146 : 121	35.8 *Vs.* 34.9	17.6 ± 6 *Vs* 18.48 ± 5.5	NA	Copper	Electronic waste

*Ca* Calcium, *HYP* hydroxyproline, *NA* Not available, *OC* osteocalcine, *PTH* parathyroid hormone, *UDP* Urinary deoxypyridinoline, *Vit-D* Vitamin D.

*Studies reporting significant difference between the occupationally Pb exposed group and control group.

### Risk of bias

Details of risk of bias related to participant selection, ascertainment of exposure and outcome parameters in individuals primary studies as per NOS is available at Table 2^25^. Briefly, notable risk for bias during participant selection included convenient sampling, inadequate representativeness of the exposed and control participants (in view of absence of precise records on exposure), non-description of precise source of control participants in some of the studies^7,9,28^ and incomplete description of control participants (i.e. occupation, confirmation of their non-exposure)^4,7,9,28^. Additionally, as the interviewer(s) were non-blinded to the participant status (as either exposed or unexposed), the exposure assessment is at risk being potentially biased. Therefore, none of the included studies was comparable to a well-conducted RCT.

**Table 2:** Newcastle Ottawa scale for assessing the risk of bias in the included studies.

	Adequacy of case definition	Case representativeness	Selection of controls	Definition of controls	Comparability of groups	Ascertainment of exposure	Similarity in the method of ascertainment	Non-response rate	Total*
Akbal et al.^5^	*	–	*	*	*	*	–	–	5
Anetor et al.^3^	–	–	*	*	*	–	*	–	4
Anetor et al.^6^	–	–	*	*	**	–	*	–	5
Batra et al.^4^	–	–	*	–	**	–	*	–	4
Dongre et al.^7^	–	–	–	–	–	–	*	–	1
Estela Kristal Boneh et al.^8^	–	*	*	*	–	–	*	*	5
Himani et al.^28^	–	–	–	–	*	–	*	–	2
Mazumder et al.^29^	–	–	*	*	–	–	*	–	3
Osterode et al.^9^	–	–	–	–	–	–	*	–	1
Pizent et al.^14^	–	*	*	*	*	–	*	*	6
Wang et al.^13^	–	–	*	*	**	–	*	–	5

*Total number of stars obtained.

### Certainty of evidence

GRADE assessment for the certainty of evidence revealed very low evidence for the association between occupational Pb exposure and all of the included calcium homeostasis markers. Detailed summary of GRADE assessment is available in the supplementary material (appendix table 5). Briefly, the risk of bias, publication bias and other potential sources of bias were potentially high considering the NOS assessment, funnel plot and contour enhance funnel plot. Further, considering the relative narrow estimate of mean difference and wide 95% confidence interval (not significant), the certainty of evidence for none of the outcome variables qualified to either moderate or high.

### Meta-analysis results of individual outcome parameters

#### Blood lead levels

**Figure 2:**
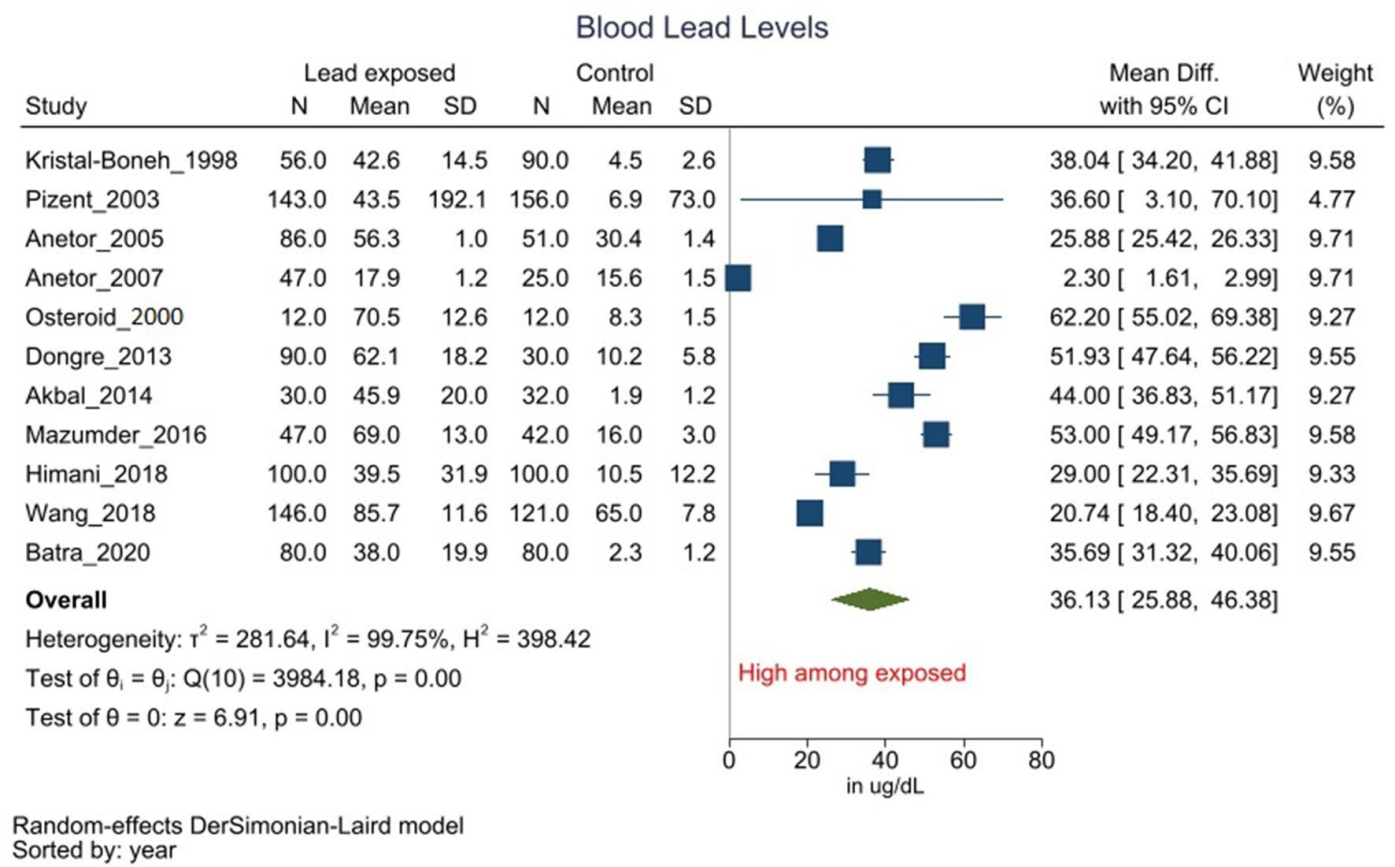
Forest plot for association between occupational Pb exposure and Blood lead levels. (Legends/footnotes): forest plot revealing the group mean differences of blood lead levels (BLL) in µg/dl between the occupationally Pb exposed workers and control participants (i.e. without obvious Pb exposure). The square and whisker (horizontal lines) represent respectively the mean difference and 95% confidence interval of individual studies. The length and width of the diamond indicate the pooled mean difference and 95% confidence interval derived from random-effect analysis.

Eleven studies reported BLL among occupationally Pb exposed & control workers^3–9,13,14,28,29^. Occupationally Pb exposed group from all included studies had significantly (*P* < 0.05) higher BLL as compared to control subjects. These individual observations were consistent with the pooled analysis, wherein occupationally Pb exposed workers had higher mean BLL of 36.13 µg/dl (95% CI 25.88 to 46.38, *I*^2^ = 99.75%, *P* < 0.05) as compared to control group (Fig. 2). Subgroup analysis of studies involving Pb exposed workers with history of additional exposure to other heavy metal(s) did not influence the overall results (Supplement Fig. 1). Explorative uni-variate meta-regression with participants’ mean age as the moderator did not significantly reduce the heterogeneity/change the results. Further, the assymteric funnel plot is suggestive of bias due to publication (*P* = 0.425), while contour enhanced funnel plot indicate the presence of additional biases. (Supplement Fig. 2).

#### Urinary lead levels

Two of the included studies reported Urine Pb levels. Pooled mean difference in urinary Pb levels between the groups was 10.89 µg/dl (95% CI − 1.01 to 22.79, *I*^2^ = 96.71). Additional analyses such as subgroup, meta-regression, sensitivity and funnel plot analyses were not executed in view of fewer studies.

#### Serum calcium

**Figure 3:**
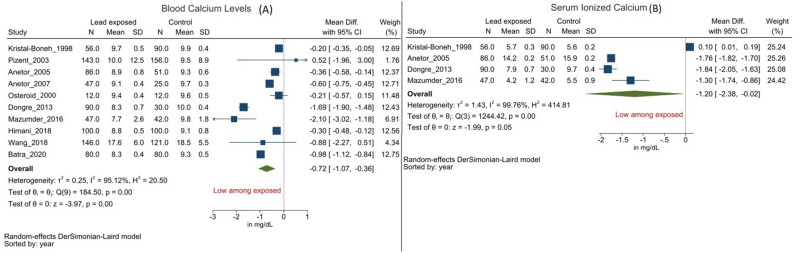
Forest plot for association between occupational Pb exposure and blood calcium levels. (Legends/footnotes): forest plot revealing the group mean differences in serum calcium (**A**) and ionised calcium (**B**) levels in mg/dl between the occupationally Pb exposed workers and control participants. The square and whisker (horizontal lines) represent respectively the mean difference and 95% confidence interval of individual studies. The length and width of the diamond indicate respectively the pooled mean difference and 95% confidence interval derived from random-effect analysis.

Ten studies reported serum calcium levels, seven of them observed significantly lower calcium levels among Pb exposed group as against the control group^3,4,6–9,13,14,28,29^. The Pb exposed group exhibited − 0.72 mg/dl (95% CI − 0.36 to − 1.07, *I*^2^ = 95.12%) lower calcium as compared to the comparative group (Fig. 3A). Subgroup analysis of studies involving Pb exposed participants with a history of additional exposure to other heavy metal(s), did not significantly reduce the heterogeneity, however reduced the magnitude of the difference between the duo (− 0.39 mg/dl, 95% CI − 0.64 to − 0.15, *I*^2^ = 72.04%)^3,6,8,13,14^ (Supplement Fig. 3). Meta-regression analysis with Participant’s mean age as moderator, did not alter the overall results. The assymetric funnel plot was indicative of publication bias (*P* = 0.779) and the contour enhanced funnel plot indicated additional biases as well (Supplement Fig. 4).

**Figure 4:**
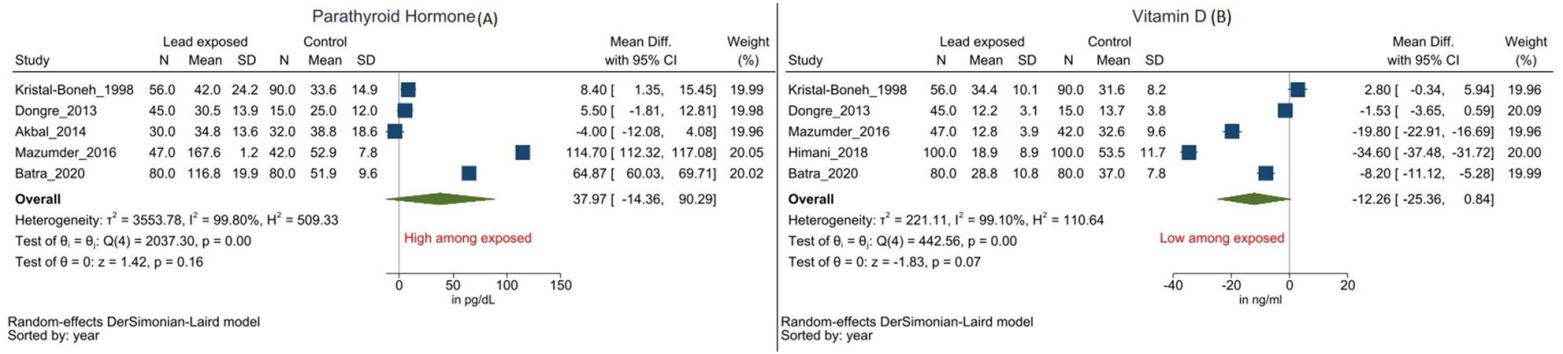
Forest Plot for parathormone (**A**) and Vitamin D (**B**). (Legends/footnotes) Group differences in paratharmone (**A**) and vitamin D (**B**) between the occupationally Pb exposed and unexposed control workers. Forest plot for association between occupational Pb exposure and Paratharmone (**A**) and Vitamin D (**B**) levels. (Legends/footnotes): Forest plot revealing the group mean differences in parathormone in pg/dl (**A**) and vitamin D in ng/dl (**B**) between the occupationally Pb exposed workers and control participants. The square and whisker (horizontal line) represent respectively the mean difference and 95% confidence interval of individual studies. The length and width of the diamond indicate respectively the pooled mean difference and 95% confidence interval derived from random-effect analysis.

#### Serum ionized Calcium

Four of the included studies reported ionized serum calcium levels. All but one study reported statistically significant lower ionized calcium levels among occupationally Pb exposed group as compared to control group^3,7,8,29^. The results were consistent with that of the serum calcium levels, i.e., the pooled mean difference revealed lower ionized calcium levels among Pb exposed group as against the control group (− 1.20 mg/dl with 95% CI − 2.38 to − 0.02 and *I*^2^ = 99.76%) (Fig. 3B). Additional analyses such as subgroup, meta-regression and funnel plot analyses were not executed in view of fewer studies.

#### Parathyroid hormone (PTH) (Paratharmone)

Five included studies reported parathormone levels; all but one observed lower serum PTH levels among Pb exposed as compared to the control group^4,5,7,8,29^. Occupationally Pb exposed group revealed mean 37.97 pg/ml (95% CI − 14.36 to 90.29 and *I*^2^ = 99.80%) lower parathormone level as compared to the control group, with unacceptable levels of heterogeneity (Fig. 4A). However, the results were statistically not significant. Subgroup and meta-regression analyses weren’t taken up, given fewer available studies reporting parathormone levels.

#### Vitamin D

Five studies reported Vitamin D levels, and all but one reported significantly lower Vitamin D levels among Pb exposed group as against the control group^4,5,7,8,28,29^. The pooled mean difference between the duo was − 12.26 (95% CI − 25.36 to 0.84, *I*^2^ = 99.10%) ng/dl with high heterogeneity (Fig. 4B). Although, the point estimate of the effect measure for Vitamin D was lower among occupationally Pb exposed, the result was statistically not significant. Subgroup and meta-regression analyses weren’t taken up, given fewer available studies reporting vitamin D levels.

#### Calcitriol

Two of the included studies reported differences in calcitriol levels (active form of vitamin D), with one study reporting higher^8^ while the other^5^ reported contrastingly lower serum calcitriol levels among occupationally Pb exposed group as against the comparative group. The pooled mean difference between the duo was − 5.98 (95% CI − 8.40 to 20.35, *I*^2^ = 91.20%) pg/dl with high heterogeneity. The results are suggestive of lower calcitriol among Pb-exposed as against the comparative group; however, the result was statistically not significant. Due to a limited number of studies, subgroup and meta-regression analysis were not taken.

#### Other outcome parameters

Akbar et al. (2014) alone compared serum osteocalcin (bone resorption marker), urinary deoxypyridinoline, and urinary hydroxyproline values between the two groups^5^. Both bone turnover markers in urine (deoxypyridinoline and hydroxyproline) and bone resorption markers (osteocalcin) were higher among the occupationally Pb exposed as compared to the control group; however, the results of urinary bone turnover markers (deoxypyridinoline and hydroxyproline) were statistically significant (*P* < 0.05).

## Discussion

Present study systematically pooled existing evidence to determine the association between chronic Pb exposure and calcium homeostasis markers. Therefore studies comparing calcium homeostasis markers among occupationally Pb exposed workers as against unexposed control participants were systematicaly reviewed. Salient observations include, chronic Pb exposure (mostly occupational) was associated with derangement of calcium homeostasis markers. However, these observations were derived by pooling relatively heterogeneous studies.

Occupationally Pb exposed workers exhibited significantly higher lead levels in blood as compared to control participants (i.e. those without a history of obvious Pb exposure). Majority of the primary studies included in the review irrespective of the type of workplace employed or the duration of exposure support observations of current sysematic review. Interestingly, the control group without obvious occupational Pb exposure too exhibited Pb in their blood, however at levels significantly lower than those with occupational Pb exposure. As Pb has no known physiological functions, the recommendations on safe BLL requires further clarity. The average reference for acceptable BLLs among the general population was earlier 1.2 µg/dl^30^, however based on observations from the “Adult Blood Lead Epidemiology and Surveillance” program, the Center for Disease Control had relaxed reference BLL to < 10 µg/dl for the general population (i.e. adults without occupational Pb exposure)^31^. Surprisingly six of the eleven studies included in present systematic review, reported mean BLL > 10 µg/dl among the unexposed control group^3,6,7,13,29^. Persistence of Pb in environment due to Pb based paints, Pb plumbings, leaded fuel and other sources could have been the potential sources of exposure and responsible for such high BLL observations among the control group. Considering the potential hazardous nature of Pb and no known physiological role, the possible sources of environmental Pb exposure should be meticulously investigated and control strategies to be employed.

Pooled results suggest the occupational Pb exposure was significantly associated with lower serum calcium (total Calcium and ionized Calcium) and a trend of higher parathormone and lower vitamin D levels. Current observations require cautious interpretation, in view of results being synthesized by pooling relatively heterogeneous primary literature (unacceptably high heterogeneity i.e., *I*^2^ > 95%), probably influenced by publication biases as inferred by the funnel plots. Lastly, for fewer primary studies available in this regard, meta-regression, subgroup, and sensitivity analyses were restricted.

Lead, a biochemical analogue of calcium, was posited to impair the activation of vitamin D (i.e., formation of calcitriol) and thereby perturb calcium metabolism (i.e., reduction in serum calcium)^3,4^. Parathormone, known to regulate the serum calcium levels by feedback mechanisms, is elevated in response to reduced calcium levels. Consistent with the posited mechanisms, occupationally Pb exposed individuals exhibited significantly lower serum calcium levels and trend of higher parathormone levels and lowered vitamin D levels in the present study. Based on current observations and previous inferences confirming the association between chronic Pb exposure and reduced prolactin levels, Pb may be regarded as an endocrine disruptor^32^.

Observations from the current study is perhaps the earliest to confirm the association between occupational Pb exposure and derangement in calcium homeostasis markers (particularly calcium) by systematically reviewing the literature and executing pooled quantitative analysis. However, the limitations inherent to systematically pooling results from primary literature such as heterogeneity among them, absence of high-powered studies, non-uniform reporting of results, and other unknown factors, should be considered while interpreting the current results. Further, the primary studies included in the present systematic review are of cross sectional observational designs, with inherent high levels of risk of bias, low – very low certainty, fewer availability of primary literature limiting the sub-group, sensitivity and meta-regression analysis and mean differences with wide confidence intervals.

Current observations hint the need for longitudinal evaluation (multiple time points) of chronically (occupationally) Pb exposed individuals for changes in levels of calcium homeostasis markers to confirm their association. Additionally, future interventional studies investigating the role of vitamin D intervention in alleviating the aberrations in calcium homeostasis markers and bone turnover/resorption markers among chronic Pb exposed individuals would generate more insight in this regard. In view of the vitality of the trace element (Calcium) in physiologic functions (viz. musculoskeletal system and second messenger) and the influence of parathormone on bones (calcium mobilization to regulate serum calcium), current study recommends their periodic evaluation among occupationally Pb exposed workers. Further, serum Pb levels may be considered as one among the potential causes, while investigating causes for chronic low calcium levels in community adults.

### Conclusion

Current results support the inverse relationship between chronic Pb exposure and calcium homeostasis markers (particularly reduction in serum calcium). However, considering the lack of evidence from high-quality studies, further evidences from multicentred longitudinal studies, including additional investigations (vitamin D metabolites, bone turnover/ resorption markers) is warranted for a precise understanding of the relation between the duo. Present observations suggest the need to draft regulations for periodic examination of calcium homeostasis markers among those chronically exposed to lead.

## Supplementary Information

Supplementary Information.
