# Supplementary material for: Association between blood lead levels and markers of calcium homeostasis: a systematic review and meta-analysis
Source: Sci Rep. 2022 Feb 3;12:1850. doi: 10.1038/s41598-022-05976-4 (PMC8814138; doi:10.1038/s41598-022-05976-4)

Supplementary Material

- Supplementary Figures
- Appendix / supplementary tables

| Supplementary Figure 1: Forest plot for the subgroup analysis of studies evaluating Blood lead levels |
| --- |
| 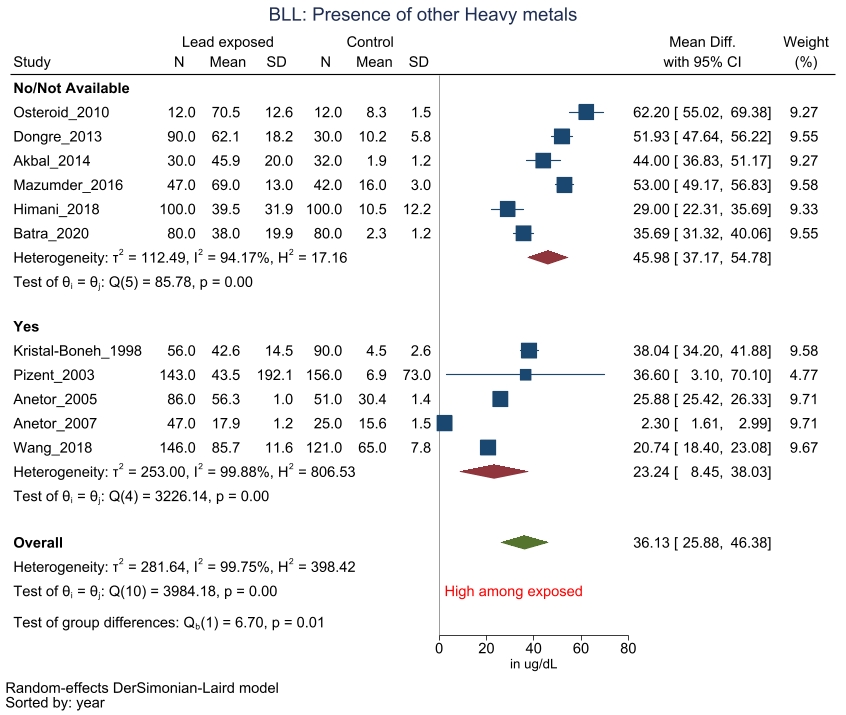  2000  2000 |
| (Legends / footnotes) Sub group analysis evaluating the influence of simultaneous exposure of additional heavy metals. |

| Supplementary Figure 2: Funnel plot and contour enhanced plot for studies evaluating BLL | |
| --- | --- |
| 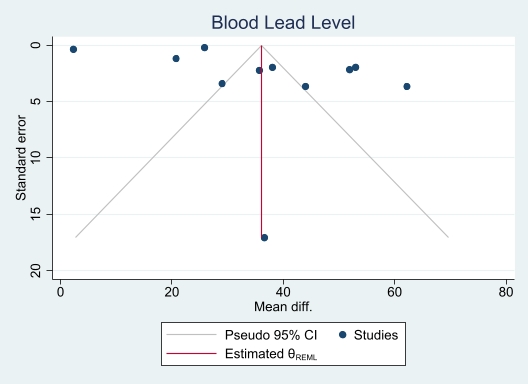 | 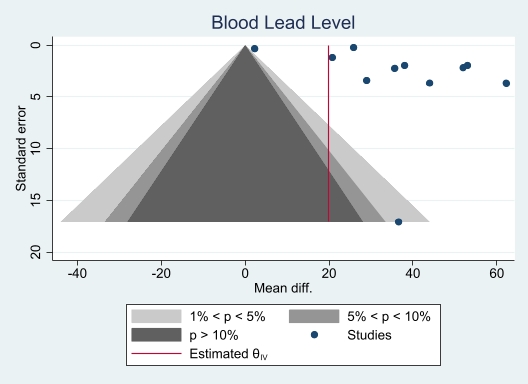 |
| (Legend / footnote) X axis represents the estimated mean difference in BLL between the 2 groups and y axis represents the precision of the measure (standard error). The (two-sided) statistical significance of any point on a funnel plot can be calculated by considering the estimated measure and precision. Various shades in the Contour - enhanced funnel plot (B) represent the levels of statistical significance (from inside -out > 10% , 1- 5 % & <1%). | |

| Supplementary Figure 3: Forest plot for the subgroup analysis of studies evaluating Calcium |
| --- |
| 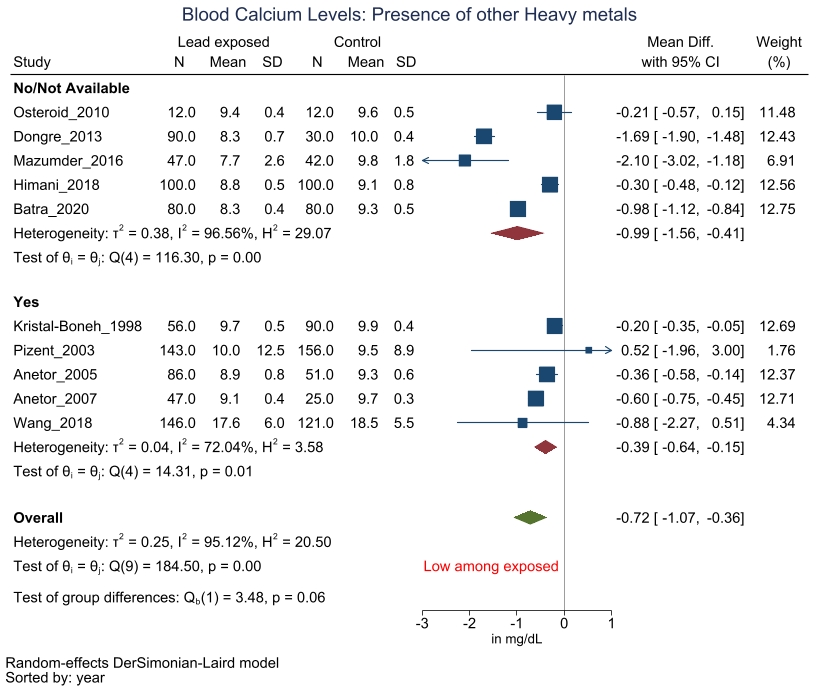  2000 |
| (Legends / footnotes) Sub group analysis evaluating the influence of simultaneous exposure of additional heavy metals. |

| Supplementary Figure 4: Funnel plot and contour enhanced plot for studies evaluating calcium | |
| --- | --- |
| 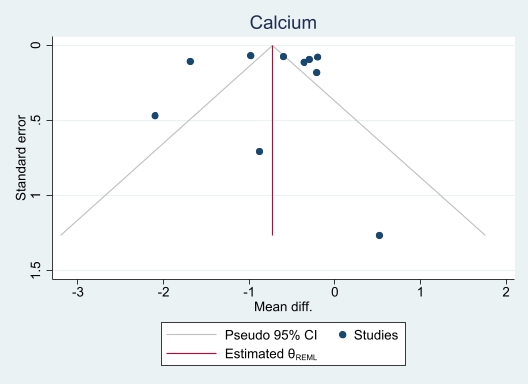 | 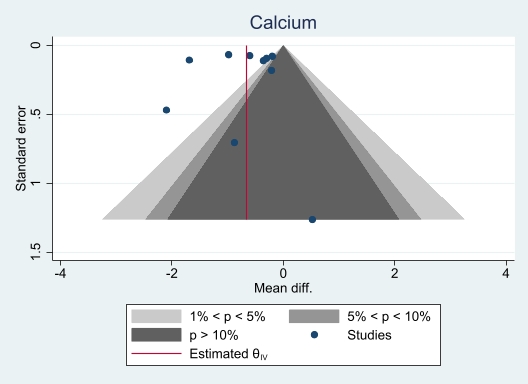 |
| (Legend / footnote) X axis represents the estimated mean difference in serum / blood calcium between the 2 groups and y axis represents the precision of the measure (standard error). The (two-sided) statistical significance of any point on a funnel plot can be calculated by considering the estimated measure and precision. Various shades in the Contour - enhanced funnel plot (B) represent the levels of statistical significance (from inside -out > 10% , 1- 5 % & <1%). | |

**Appendix**

Supplementary table 1: Search strategy at Pubmed-medline repository

| PICOS | Pubmed-Medline |
| --- | --- |
| Intervention / exposure | “lead acetate” OR “lead tetraoxide” OR “lead tetraacetate” OR “lead chromate” OR “lead phosphate” OR “lead oxide”OR “lead silicate” OR “tetraethyl lead” OR lead[MeSH Terms] OR “lead poisoning” OR “Pb” OR “plumbum” OR “tetraethyl Lead” |
| Outcome | **Calcium:**  Calcium |
|  | **Parathyroid:**  "Parathyroid Hormone"[Mesh] OR PTH OR parathyroid OR 'parathormon' OR 'parathyrin' |
|  | **Vitamin D:**  Vit D OR Vitamin D OR calcitriol OR Cholecalciferol OR ergocalciferol OR “25-hydroxyvitamin D” OR “25(OH) D” OR “1,25-dihydroxyvitamin D2” OR 1,25-dihydroxyvitamin D3 OR 1,25-dihydroxyvitamin D |
|  | **Calcitonin**:  Calcitonin OR thyrocalcitonin OR Procalcitonin OR secretin OR "Parathyroid Hormone"[Mesh] OR parathyroid |
|  | Lead AND Calcium |
|  | Lead AND Parathyroid |
|  | Lead AND Vitamin D |
|  | Lead AND Calcitonin |

Supplementary table 2: Search strategy used at Embase database:

| PICOS | EMBASE |
| --- | --- |
| Intervention / exposure | 'lead'/exp OR '208pb' OR 'inorganic lead' OR 'lead' OR 'lead 208' OR 'lead ion' OR 'lead ore' OR 'lead radioisotope' OR 'lead radioisotopes' OR 'plumbum' OR 'radioisotope, lead' OR 'lead acetate'/exp OR 'lead acetate' OR 'lead acetate medium' OR 'lead diacetate' OR 'neutral lead acetate' OR 'lead tetraoxide'/exp OR 'lead chromate'/exp OR 'c.i. pigment yellow 34' OR 'chrome yellow' OR 'cologne yellow' OR 'kings yellow' OR 'lead chromate' OR 'leipzig yellow' OR 'paris yellow' OR 'lead phosphate'/exp OR 'lead oxide'/exp OR 'lead monoxide' OR 'lead oxide' OR 'lead oxide yellow' OR 'lead protoxide' OR 'litharge' OR 'massicot' OR 'plumbous oxide' OR 'lead silicate' OR 'tetraethyllead'/exp OR 'lead tetraethyl' OR 'tetra ethyl lead' OR 'tetraethyl lead' OR 'tetraethyllead' |
| Outcome | **Calcium:**  'calcium'/exp |
|  | **Parathyroid:**  'parathyroid gland hormone'/exp OR 'parathyroid gland hormone' OR 'parathyroid hormone'/exp OR 'pth' OR 'bovine parathyroid hormone' OR 'human parathyroid hormone' OR 'parathorm' OR 'parathormon' OR 'parathormone' OR 'parathyrin' OR 'parathyroid hormone' OR 'parathyroid hormone 1 84' OR 'parathyroid hormone 1-84' OR 'parathyroid hormone [1-84]' OR 'parathyroid hormones' |
|  | **Vitamin D:**  'vitamin d'/exp OR 'vitamin d' OR 'calcitriol'/exp OR '1 alpha 25 dihydroxyvitamin d 3' OR '1, 25 dihydrocholecalciferol' OR '1, 25 dihydroxy cholecalciferol' OR '1, 25 dihydroxy vitamin d 3' OR '1, 25 dihydroxycalciferol' OR '1, 25 dihydroxycholecalciferol' OR '1, 25 dihydroxycolecalciferol' OR '1, 25 dihydroxyvitamin d' OR '1, 25 dihydroxyvitamin d 3' OR '1, 25 dihydroxyvitamin d3' OR '1-alfa, 25-dihydroxycholecalciferol' OR '1alpha 25 dihydroxycolecalciferol' OR '1alpha, 25 dihydroxycholecalciferol' OR '1alpha, 25 dihydroxycolecalciferol' OR '1alpha, 25 dihydroxyvitamin d 3' OR '1alpha, 25 dihydroxyvitamin d3' OR '9, 10 secocholesta 5, 7, 10 (19) triene 1alpha, 3beta, 25 triol' OR 'bocatriol' OR 'bonky' OR 'cabone' OR 'calcijex' OR 'calcitriol' OR 'caraben sc' OR 'cholecalciferol, 1alpha, 25 dihydroxy' OR 'cicarol' OR 'citrihexal' OR 'colecalciferol 1, 25 diol' OR 'colecalciferol, 1alpha, 25 dihydroxy' OR 'decostriol' OR 'difix' OR 'dn 101' OR 'dn101' OR 'ecatrol' OR 'ecatrol f' OR 'hitrol' OR 'kolkatriol' OR 'kosteo' OR 'lemytriol' OR 'meditrol' OR 'osteotriol' OR 'poscal' OR 'renatriol' OR 'rexamat' OR 'ro 21 5535' OR 'rocaltrol' OR 'roical' OR 'rolsical' OR 'silkis' OR 'sitriol' OR 'soltriol' OR 'tariol' OR 'tirocal' OR 'triocalcit' OR 'vectical' OR 'vitamin d3, 1, 25 dihydroxy' OR 'ergocalciferol'/exp OR '24 methyl 9, 10 secocholesta 5, 7, 10 (19), 22 tetraen 3 ol' OR '9, 10 seco 5, 7, 10 (19), 22 ergostatetraen 3beta ol' OR '9, 10 secoergosta 5, 7, 10 (19), 22 tetraen 3 ol 19, 22 tetraen 3 ol' OR 'afj d2' OR 'alcovit d2' OR 'aldevit' OR 'bentavit' OR 'calciferol' OR 'calciferovit' OR 'chemovit d' OR 'chocola d' OR 'condol' OR 'd arthrin' OR 'd arthrine' OR 'd crivit' OR 'd vatine' OR 'd vital' OR 'd2 vita' OR 'davitamon d' OR 'davitan' OR 'davitin' OR 'decaps' OR 'dee osterol' OR 'dee ron' OR 'deeosterol' OR 'deeron' OR 'dekristol' OR 'delta monovit' OR 'deltabios' OR 'deltalin' OR 'deltaline' OR 'deltamonovit' OR 'deltar' OR 'deltasterolo' OR 'deltavit' OR 'deradion' OR 'deradione' OR 'deratol' OR 'dergosten' OR 'desyn' OR 'desyne' OR 'detalup' OR 'detamine' OR 'deterapion' OR 'deterapione' OR 'devitan' OR 'devitil' OR 'devitol' OR 'di actol' OR 'di drol' OR 'diactol' OR 'dibiovit' OR 'didrol' OR 'didue vita' OR 'diergin' OR 'diergine' OR 'diferol' OR 'difilina' OR 'difvitamin d' OR 'dilavit' OR 'disir' OR 'disterina' OR 'disterine' OR 'divit urto' OR 'divitina' OR 'divitine' OR 'diviturto' OR 'dohyfral d' OR 'drisdol' OR 'dumovit d' OR 'dz idrosol' OR 'endo d' OR 'ercalciol' OR 'ergocalciferol' OR 'ergocalciferols' OR 'ergorone' OR 'ergosterid' OR 'ergosteride' OR 'ergosterin activatum' OR 'ergosterina irradiata' OR 'ertron' OR 'ertrone' OR 'feroxyl' OR 'feroxyle' OR 'fortedol' OR 'fortodyl' OR 'fortodyle' OR 'geltabs vitamin d' OR 'genevis' OR 'glicol d2' OR 'idro steral' OR 'idrosol d2' OR 'infadin' OR 'infadine' OR 'infron' OR 'infrone' OR 'inovitan d' OR 'irradiated ergosterol' OR 'kalciferol' OR 'metadee' OR 'mina d2' OR 'mine d2' OR 'mulsiferol' OR 'mykostin' OR 'mykostine' OR 'oldevit' OR 'oleovit d2' OR 'oleovitamin d2' OR 'ostelin' OR 'osteline' OR 'osteodina' OR 'osteodine' OR 'osteovit' OR 'osteovitadin' OR 'osteovitadine' OR 'osteovitina' OR 'osteovitine' OR 'ostergil' OR 'plivit d' OR 'radiamon' OR 'radiosterina' OR 'radiosterine' OR 'radiostol' OR 'radsterin' OR 'radsterine' OR 'raquiferol' OR 'raquiferol d3' OR 'ro 850' OR 'shock ferol' OR 'shockferol' OR 'sinervit d2' OR 'steral' OR 'steramin' OR 'steramine' OR 'sterobiol' OR 'sterodin' OR 'sterodine' OR 'sterogyl' OR 'sterogyl 15' OR 'sterogyl-15' OR 'sterosol' OR 'sterovit' OR 'sterovitina' OR 'sterovitine' OR 'ucemine d' OR 'ultranol' OR 'urto calciosterina' OR 'urtosterina' OR 'urtosterine' OR 'uvesterol d' OR 'vi de' OR 'vi di' OR 'vide' OR 'videlta' OR 'vidi' OR 'vidiman' OR 'vidolen' OR 'vidue monico' OR 'viduemonico' OR 'vigoncal' OR 'vio d' OR 'viosterin' OR 'viosterine' OR 'viosterol' OR 'vitadit' OR 'vitamin d2' OR 'vitamin d 2' OR 'vitaminol' OR 'vitaplex' OR 'vitasan d' OR 'vitastabil d' OR 'vitastabile d' OR 'vitasterin' OR 'vitasterine' OR 'vitasterol' OR 'vitavel d' OR 'wandervit d2' |
|  | **Calcitonin**:  'calcitonin'/exp OR 'alpha calcitonin' OR 'alpha thyrocalcitonin' OR 'beta thyrocalcitonin' OR 'calcitar' OR 'calcitocin' OR 'calcitonia' OR 'calcitonin' OR 'calcitonin [1-32]' OR 'calcitonin binding protein' OR 'calcitonin human' OR 'calcitonin m' OR 'calcitonin monomer' OR 'calcitonin release' OR 'calcitonin secretion' OR 'calcitonine' OR 'cibacalcin' OR 'cibalcin' OR 'human calcitonin' OR 'porcine calcitonin' OR 'staporos' OR 'thyreocalcitonine' OR 'thyrocalcitonin' OR 'thyrocalcitonine' OR 'secretin'/exp OR 'chirhostim' OR 'hoe 069' OR 'hoe069' OR 'human secretin' OR 'rg 1068' OR 'rg1068' OR 'secreflo' OR 'secremax' OR 'secrepan' OR 'secretin' OR 'secretin human' OR 'secretin pentahydrochloride' OR 'secretin synthetic human' OR 'secretin synthetic porcine' OR 'secretin-ferring' OR 'secretine' OR 'secretolyn' OR 'sekretolin' OR 'synthetic secretin' |
|  | *Lead AND Calcium (only humans, journal articles, adolescents and above age)* |
|  | *Lead AND Parathyroid (only humans & journal articles)* |
|  | *Lead AND Vitamin D (only humans & journal articles)* |
|  | *Lead AND Calcitonin* *(only humans & journal articles)* |

Supplementary table 3: Search results at scopus database

| PICOS | Scopus |
| --- | --- |
| Intervention / exposure | "lead acetate" OR "lead tetraoxide" OR "lead tetraacetate" OR "lead chromate" OR "lead phosphate" OR "lead oxide" OR "lead silicate" OR "tetraethyl lead" OR "lead poisoning" OR "Pb" OR "plumbum" OR "tetraethyl" OR TITLE ( "lead " ) OR ABS ( "Pb" ) |
| Outcome | **Calcium:**  ( TITLE-ABS-KEY ( ( serum OR blood OR urine ) AND "calcium" ) |
|  | **Parathyroid:**  "Parathyroid Hormone" OR PTH OR parathyroid OR 'parathormon' OR 'parathyrin' |
|  | **Vitamin D:**  “Vit D” OR “Vitamin D” OR calcitriol OR Cholecalciferol OR ergocalciferol OR “25-hydroxyvitamin D” OR “25(OH) D” OR “1,25-dihydroxyvitamin D2” OR “1,25-dihydroxyvitamin D3” OR “1,25-dihydroxyvitamin D” |
|  | **Calcitonin**:  Calcitonin OR thyrocalcitonin OR Procalcitonin OR secretin OR "Parathyroid Hormone" OR parathyroid |
|  | Lead AND Calcium (Limit to Human, Journal article) |
|  | Lead AND Parathyroid (Limit to Human, Journal article) |
|  | Lead AND Vitamin D (Limit to Human, Journal article) |
|  | Lead AND Calcitonin (Limit to Human, Journal article) |

Supplementary table 4 List of articles excluded during full text screening along with the reasons (a particular study could have more than one of the listed reasons for exclusion)

| Sr. No. | Citation | Reason for exclusion |
| --- | --- | --- |
|  | Adachi, Arlen et al. 1998(1) | Wrong population* |
|  | Afridi, Talpur et al. 2015(2) | Outcome of interest not available^@^ |
|  | Aguilar-Dorado, Hernández et al. 2014(3) | Outcome of interest not available^@^ |
|  | Akbal, Tutkun et al. 2014(4) | NA (included)^#^ |
|  | Alba, Carleton et al. 2012(5) | Review |
|  | Al-Ghafari, Elmorsy et al. 2019(6) | Wrong population* |
|  | Anetor, Adeniyi et al. 1999(7) | Wrong population* |
|  | Anetor, Ajose et al. 2007(8) | NA (included) |
|  | Anetor, Akingbola et al. 2005(9) | NA (included) |
|  | Antonowicz, Andrzejczak et al. 1991(10) | Outcome of interest not available^@^ |
|  | Aoki, Brody et al. 2016(11) | Wrong population* |
|  | Araki, Aono et al. 1986(12) | Outcome of interest not available^@^ |
|  | Araki and Ushio 1982(13) | Outcome of interest not available^@^ |
|  | Balani, Golla et al. 2016(14) | Case study |
|  | Bartolozzi and Zurlo 1960(15) | Wrong population* |
|  | Batra, Thakur et al. 2020(16) | NA (included) |
|  | Campbell and Auinger 2007(17) | Wrong population* |
|  | Chalkley, Richmond et al. 1998(18) | Wrong population* |
|  | Chuang, Yu et al. 2004(19) | Wrong population* |
|  | Dobrakowski, Boron et al. 2017(20) | Wrong population* |
|  | Dongre, Suryakar et al. 2013(21) | NA (included) |
|  | Ettinger, Lamadrid-Figueroa et al. 2009(22) | Wrong population* |
|  | Ghosh-Narang, Jones et al. 2007(23) | Wrong population* |
|  | Himani, Kumar et al. 2020(24) | NA (included) |
|  | Hu, Aro et al. 1996(25) | Wrong population* |
|  | Jackson, Cromer et al. 2010(26) | Wrong population* |
|  | Kristal-Boneh, Froom et al. 1998(27) | NA (included) |
|  | Lee and Kim 2012(28) | Wrong population* |
|  | Liu, Zhang et al. 2019(29) | Outcome of interest not available^@^ |
|  | Mason, Somervaille et al. 1990(30) | Outcome of interest not available^@^ |
|  | Mazumdar, Goswami et al. 2017(31) | NA (included) |
|  | Meredith, Campbell et al. 1977(32) | Wrong population* |
|  | Morris, McCarron et al. 1990(33) | Others^$^ |
|  | Muldoon, Cauley et al. 1994(34) | Wrong population* |
|  | Osterloh and Clark 1993(35) | Others^$^ |
|  | Osterode and Ulberth 2000(36) | NA (included) |
|  | Pizent, Jurasović et al. 2003(37) | NA (included) |
|  | Potula, Henderson et al. 2005(38) | Wrong population* |
|  | Quintanar-Escorza, González-Martínez et al. 2007(39) | Outcome of interest not available^@^ |
|  | Riedt, Buckley et al. 2009(40) | Wrong population* |
|  | Sata, Araki et al. 1998(41) | Others^$^ |
|  | Shaik and Jamil 2009(42) | Wrong population* |
|  | Sharp, Beswick et al. 1991(43) | Wrong population* |
|  | Theppeang, Glass et al. 2008(44) | Wrong population* |
|  | Thomasino, Zuroweste et al. 1977(45) | Wrong population* |
|  | Truckenbrodt, Winter et al. 1984(46) | Wrong population* |
|  | Wang, Sun et al. 2018(47) | NA (included) |
|  | Weaver, Lee et al. 2006(48) | Wrong population* |
|  | Weyermann and Brenner 1997(49) | Wrong population* |
|  | Weyermann and Brenner 1998(50) | Wrong population* |
|  | Yang, Wu et al. 2012(51) | Wrong population* |
|  | Zeqiri, Zeqiri et al. 2012(52) | Wrong population* |

* Wrong population included studies those involved either participants with no occupational Pb exposure or pre-existing illness or no control / comparative group or community population or sub-acute Pb exposure or similar reasons

^@^ Outcome of interest for the current study were not reported in these studies

^#^ Studies are included in the current systematic review

^$^ Reasons other than listed above such as pre-clinical models

References of records identified for full text review

1. Adachi JD, Arlen D, Webber CE, Chettle DR, Beaumont LF, Gordon CL. Is there any association between the presence of bone disease and cumulative exposure to lead? Calcified Tissue International. 1998;63(5):429-32.

2. Afridi HI, Talpur FN, Kazi TG, Kazi N, Arain SS, Shah F. Estimation of calcium, magnesium, cadmium, and lead in biological samples from paralyzed quality control and production steel mill workers. Environ Monit Assess. 2015;187(6):350.

3. Aguilar-Dorado IC, Hernández G, Quintanar-Escorza MA, Maldonado-Vega M, Rosas-Flores M, Calderón-Salinas JV. Eryptosis in lead-exposed workers. Toxicology and Applied Pharmacology. 2014;281(2):195-202.

4. Akbal A, Tutkun E, Yilmaz H. Lead exposure is a risk for worsening bone mineral density in middle-aged male workers. Aging Male. 2014;17(3):189-93.

5. Alba A, Carleton L, Dinkel L, Ruppe R. Increased lead levels in pregnancy among immigrant women. Journal of midwifery & women's health. 2012;57(5):509-14.

6. Al-Ghafari A, Elmorsy E, Fikry E, Alrowaili M, Carter WG. The heavy metals lead and cadmium are cytotoxic to human bone osteoblasts via induction of redox stress. PLoS ONE. 2019;14(11).

7. Anetor JI, Adeniyi FA, Taylor GO. Biochemical indicators of metabolic poisoning associated with lead based occupations in nutritionally disadvantaged communities. African journal of medicine and medical sciences. 1999;28(1):9-12.

8. Anetor JI, Ajose OA, Adebiyi JA, Akingbola TS, Iy, a AA, et al. Decreased thiamine and magnesium levels in the potentiation of the neurotoxicity of lead in occupational lead exposure. Biological Trace Element Research. 2007;116(1):43-51.

9. Anetor JI, Akingbola TS, Adeniyi FAA, Taylor GOI. Decreased total and ionized calcium levels and haematological indices in occupational lead exposure as evidence of the endocrine disruptive effect of lead. Indian Journal of Occupational and Environmental Medicine. 2005;9(1):15-21.

10. Antonowicz J, Andrzejczak R, Kuliczkowski K, Smolik R. Levels of trace elements in the serum and erythrocytes and some parameters of erythrocyte heme metabolism (FEP, ALA-D, ALA-U) in copper foundry workers. Polish Journal of Occupational Medicine and Environmental Health. 1991;4(4):339-48.

11. Aoki Y, Brody DJ, Flegal KM, Fakhouri TH, Axelrad DA, Parker JD. Blood Lead and Other Metal Biomarkers as Risk Factors for Cardiovascular Disease Mortality. Medicine. 2016;95(1):e2223.

12. Araki S, Aono H, Murata K. Mobilisation of heavy metals into the urine by CaEDTA: relation to erythrocyte and plasma concentrations and exposure indicators. British journal of industrial medicine. 1986;43(9):636-41.

13. Araki S, Ushio K. Assessment of the body burden of chelatable lead: a model and its application to lead workers. British journal of industrial medicine. 1982;39(2):157-60.

14. Balani A, Golla N, Dey AK, Mahankali S, Seelam S. Image of the month: Intracranial calcifications due to chronic lead exposure. Clinical Medicine, Journal of the Royal College of Physicians of London. 2016;16(5):494.

15. Bartolozzi O, Zurlo N. [Effects of prophylaxis with versenate calcium in workers exposed to lead]. Med Lav. 1960;51:607-11.

16. Batra J, Thakur A, Meena SK, Singh L, Kumar J, Juyal D. Blood lead levels among the occupationally exposed workers and its effect on calcium and vitamin D metabolism: A case-control study. J Family Med Prim Care. 2020;9(5):2388-93.

17. Campbell JR, Auinger P. The association between blood lead levels and osteoporosis among adults--results from the third national health and nutrition examination survey (NHANES III). Environmental health perspectives. 2007;115(7):1018-22.

18. Chalkley SR, Richmond J, Barltrop D. Measurement of vitamin D3 metabolites in smelter workers exposed to lead and cadmium. Occupational and environmental medicine. 1998;55(7):446-52.

19. Chuang HY, Yu KT, Ho CK, Wu MT, Lin GT, Wu TN. Investigations of vitamin D receptor polymorphism affecting workers' susceptibility to lead. Journal of occupational health. 2004;46(4):316-22.

20. Dobrakowski M, Boron M, Birkner E, Kasperczyk A, Chwalinska E, Lisowska G, et al. The Effect of a Short-Term Exposure to Lead on the Levels of Essential Metal Ions, Selected Proteins Related to Them, and Oxidative Stress Parameters in Humans. Oxidative medicine and cellular longevity. 2017;2017:8763793.

21. Dongre NN, Suryakar AN, Patil AJ, Hundekari IA, Devarnavadagi BB. Biochemical effects of lead exposure on battery manufacture workers with reference to blood pressure, calcium metabolism and bone mineral density. Indian Journal of Clinical Biochemistry. 2013;28(1):65-70.

22. Ettinger AS, Lamadrid-Figueroa H, Tellez-Rojo MM, Mercado-Garcia A, Peterson KE, Schwartz J, et al. Effect of calcium supplementation on blood lead levels in pregnancy: a randomized placebo-controlled trial. Environmental health perspectives. 2009;117(1):26-31.

23. Ghosh-Narang J, Jones TM, Menke A, Todd AC, Muntner P, Batuman V. Parathyroid hormone status does not influence blood and bone lead levels in dialysis patients. The American journal of the medical sciences. 2007;334(6):415-20.

24. Himani, Kumar R, Ansari JA, Mahdi AA, Sharma D, Karunan, et al. Blood Lead Levels in Occupationally Exposed Workers Involved in Battery Factories of Delhi-NCR Region: Effect on Vitamin D and Calcium Metabolism. Indian Journal of Clinical Biochemistry. 2020;35(1):80-7.

25. Hu H, Aro A, Payton M, Korrick S, Sparrow D, Weiss ST, et al. The relationship of bone and blood lead to hypertension. The Normative Aging Study. Jama. 1996;275(15):1171-6.

26. Jackson LW, Cromer BA, Panneerselvamm A. Association between bone turnover, micronutrient intake, and blood lead levels in pre- and postmenopausal women, NHANES 1999-2002. Environmental health perspectives. 2010;118(11):1590-6.

27. Kristal-Boneh E, Froom P, Yerushalmi N, Harari G, Ribak J. Calcitropic hormones and occupational lead exposure. American Journal of Epidemiology. 1998;147(5):458-63.

28. Lee BK, Kim Y. Association between bone mineral density and blood lead level in menopausal women: analysis of 2008-2009 Korean National Health and Nutrition Examination Survey data. Environmental research. 2012;115:59-65.

29. Liu J, Zhang L, Feng L, Xu M, Gao Y, Zhou P, et al. Association between single nucleotide polymorphism (rs4252424) in TRPV5 calcium channel gene and lead poisoning in Chinese workers. Molecular genetics & genomic medicine. 2019;7(3):e562.

30. Mason HJ, Somervaille LJ, Wright AL, Chettle DR, Scott MC. Effect of Occupational Lead Exposure on Serum 1,25-dihydroxyvitamin D Levels. Human &amp; Experimental Toxicology. 1990;9(1):29-34.

31. Mazumdar I, Goswami K, Ali MS. Status of Serum Calcium, Vitamin D and Parathyroid Hormone and Hematological Indices Among Lead Exposed Jewelry Workers in Dhaka, Bangladesh. Indian Journal of Clinical Biochemistry. 2017;32(1):110-6.

32. Meredith PA, Campbell BC, Moore MR, Goldberg A. The effects of industrial lead poisoning on cytochrome P450 mediated phenazone (antipyrine) hydroxylation. European journal of clinical pharmacology. 1977;12(3):235-9.

33. Morris C, McCarron DA, Bennett WM. Low-level lead exposure, blood pressure, and calcium metabolism. American journal of kidney diseases : the official journal of the National Kidney Foundation. 1990;15(6):568-74.

34. Muldoon SB, Cauley JA, Kuller LH, Scott J, Rohay J. Lifestyle and sociodemographic factors as determinants of blood lead levels in elderly women. Am J Epidemiol. 1994;139(6):599-608.

35. Osterloh JD, Clark OH. Effects of hyperparathyroidism on blood lead concentrations in man. Environmental research. 1993;62(1):1-6.

36. Osterode W, Ulberth F. Increased concentration of arachidonic acid in erythrocyte membranes in chronically lead-exposed men. Journal of Toxicology and Environmental Health - Part A. 2000;59(2):87-95.

37. Pizent A, Jurasović J, Telišman S. Serum calcium, zinc, and copper in relation to biomarkers of lead and cadmium in men. Journal of Trace Elements in Medicine and Biology. 2003;17(3):199-205.

38. Potula V, Henderson A, Kaye W. Calcitropic hormones, bone turnover, and lead exposure among female smelter workers. Archives of environmental & occupational health. 2005;60(4):195-204.

39. Quintanar-Escorza MA, González-Martínez MT, Navarro L, Maldonado M, Arévalo B, Calderón-Salinas JV. Intracellular free calcium concentration and calcium transport in human erythrocytes of lead-exposed workers. Toxicology and Applied Pharmacology. 2007;220(1):1-8.

40. Riedt CS, Buckley BT, Brolin RE, Ambia-Sobhan H, Rhoads GG, Shapses SA. Blood lead levels and bone turnover with weight reduction in women. Journal of exposure science & environmental epidemiology. 2009;19(1):90-6.

41. Sata F, Araki S, Murata K, Aono H. Behavior of heavy metals in human urine and blood following calcium disodium ethylenediamine tetraacetate injection: observations in metal workers. J Toxicol Environ Health A. 1998;54(3):167-78.

42. Shaik AP, Jamil K. Individual susceptibility and genotoxicity in workers exposed to hazardous materials like lead. Journal of hazardous materials. 2009;168(2-3):918-24.

43. Sharp DS, Beswick A, Renaud S, Toothill C, Elwood PC. Blood lead and platelet aggregation--evidence for a causal association. Thrombosis and haemostasis. 1991;66(5):604-8.

44. Theppeang K, Glass TA, Bandeen-Roche K, Todd AC, Rohde CA, Links JM, et al. Associations of bone mineral density and lead levels in blood, tibia, and patella in urban-dwelling women. Environmental health perspectives. 2008;116(6):784-90.

45. Thomasino JA, Zuroweste E, Brooks SM, Petering HG, Lerner SI, Finelli VN. Lead, zinc, and erythrocyte delta-aminolevulinic acid dehydratase: relationships in lead toxicity. Archives of environmental health. 1977;32(6):244-7.

46. Truckenbrodt R, Winter L, Schaller KH. [Effect of occupational lead exposure on various elements in the human blood. Effects on calcium, cadmium, iron, copper, magnesium, manganese and zinc levels in the human blood, erythrocytes and plasma in vivo]. Zentralbl Bakteriol Mikrobiol Hyg B. 1984;179(3):187-97.

47. Wang Y, Sun X, Fang L, Li K, Yang P, Du L, et al. Genomic instability in adult men involved in processing electronic waste in Northern China. Environment International. 2018;117:69-81.

48. Weaver VM, Lee BK, Todd AC, Ahn KD, Shi W, Jaar BG, et al. Effect modification by delta-aminolevulinic acid dehydratase, vitamin D receptor, and nitric oxide synthase gene polymorphisms on associations between patella lead and renal function in lead workers. Environmental research. 2006;102(1):61-9.

49. Weyermann M, Brenner H. Alcohol consumption and smoking habits as determinants of blood lead levels in a national population sample from Germany. Archives of environmental health. 1997;52(3):233-9.

50. Weyermann M, Brenner H. Factors affecting bone demineralization and blood lead levels of postmenopausal women--a population-based study from Germany. Environmental research. 1998;76(1):19-25.

51. Yang Y, Wu J, Sun P. Effects of delta-aminolevulinic acid dehydratase polymorphisms on susceptibility to lead in Han subjects from southwestern China. International journal of environmental research and public health. 2012;9(7):2326-38.

52. Zeqiri N, Zeqiri S, Skenderaj S. Blood pressure evaluation at the workers exposed to lead. Medicinski arhiv. 2012;66(2):92-3.

**Supplementary table 5: Summary of findings of GRADE Assessment**

Evidence Profile using Grading of Recommendation, Assessment, Development, and Evaluation (GRADE) instrument

**Population:** All adults

**Exposure (intervention):** Occupational lead (Pb) exposure

**Control:** No obvious exposure to Pb

**Outcomes:** Levels of calcium homeostasis markers.

| **Outcome: Lead calcium (assessed with mean differences in calcium levels using meta-analysis)** | | | | | | | | | |
| --- | --- | --- | --- | --- | --- | --- | --- | --- | --- |
| **Quality assessment** | | | | | | **Summary of findings** | | | **Comments** |
| **No of studies** | **Risk of Bias** | **Inconsistency** | **Indirectness** | **Imprecision** | **Publication Bias** | **Mean difference** | | **Certainty/Quality** |  |
|  |  |  |  |  |  | **INB** | **95%CI** |  |  |
| Serum calcium levels among occupational Pb exposed as compared to those without obvious Pb exposure (assessed with meta-analysis). | | | | | | | | | |
| 10 | Serious | Serious ^a^ | Serious | Serious ^b^ | likely | -0.72 mg/dl | (-0.36 to -1.07) | ⊕🌕🌕🌕  Very Low |  |
| Serum parathyroid levels among occupational Pb exposed as compared to those without obvious Pb exposure (assessed with meta-analysis). | | | | | | | | | |
| 5 | Serious | Serious ^a^ | Serious | Serious ^b^ | likely | -37.97 pg/dl | (-14.36 to 90.29) | ⊕🌕🌕🌕  Very Low |  |
| Serum vitamin D levels among occupational Pb exposed as compared to those without obvious Pb exposure (assessed with meta-analysis). | | | | | | | | | |
| 5 | Serious | Serious ^a^ | Serious | Serious ^b^ | likely | -12.26 ng/dl | (-25.36 to 0.84) | ⊕🌕🌕🌕  Very Low |  |
| Serum ionized calcium levels among occupational Pb exposed as compared to those without obvious Pb exposure (assessed with meta-analysis). | | | | | | | | | |
| 4 | Serious | Serious ^a^ | Serious | Serious ^b^ | likely | -1.2 mg/dl | (-2.38 to -0.02) | ⊕🌕🌕🌕  Very Low |  |
| Serum calcitriol (1, 25(OH)_2_ D_3_) levels among occupational Pb exposed as compared to those without obvious Pb exposure (assessed with meta-analysis). | | | | | | | | | |
| 2 | Serious | Serious ^a^ | Serious | Serious ^b^ | likely | -5.98 | (-8.4 to 20.35) | ⊕🌕🌕🌕  Very Low |  |

^a^ high heterogeneity *I*^2^ > 90% ^b^ studies included have reported a wide confidence intervals

**GRADE Working Group grades of evidence**
**High certainty:** we are very confident that the true effect lies close to that of the estimate of the effect.
**Moderate certainty:** we are moderately confident in the effect estimate; the true effect is likely to be close to the estimate of the effect, but there is a possibility that it is substantially different.
**Low certainty:** our confidence in the effect estimate is limited; the true effect may be substantially different from the estimate of the effect.
**Very low certainty:** we have very little confidence in the effect estimate; the true effect is likely to be substantially different from the estimate of effect


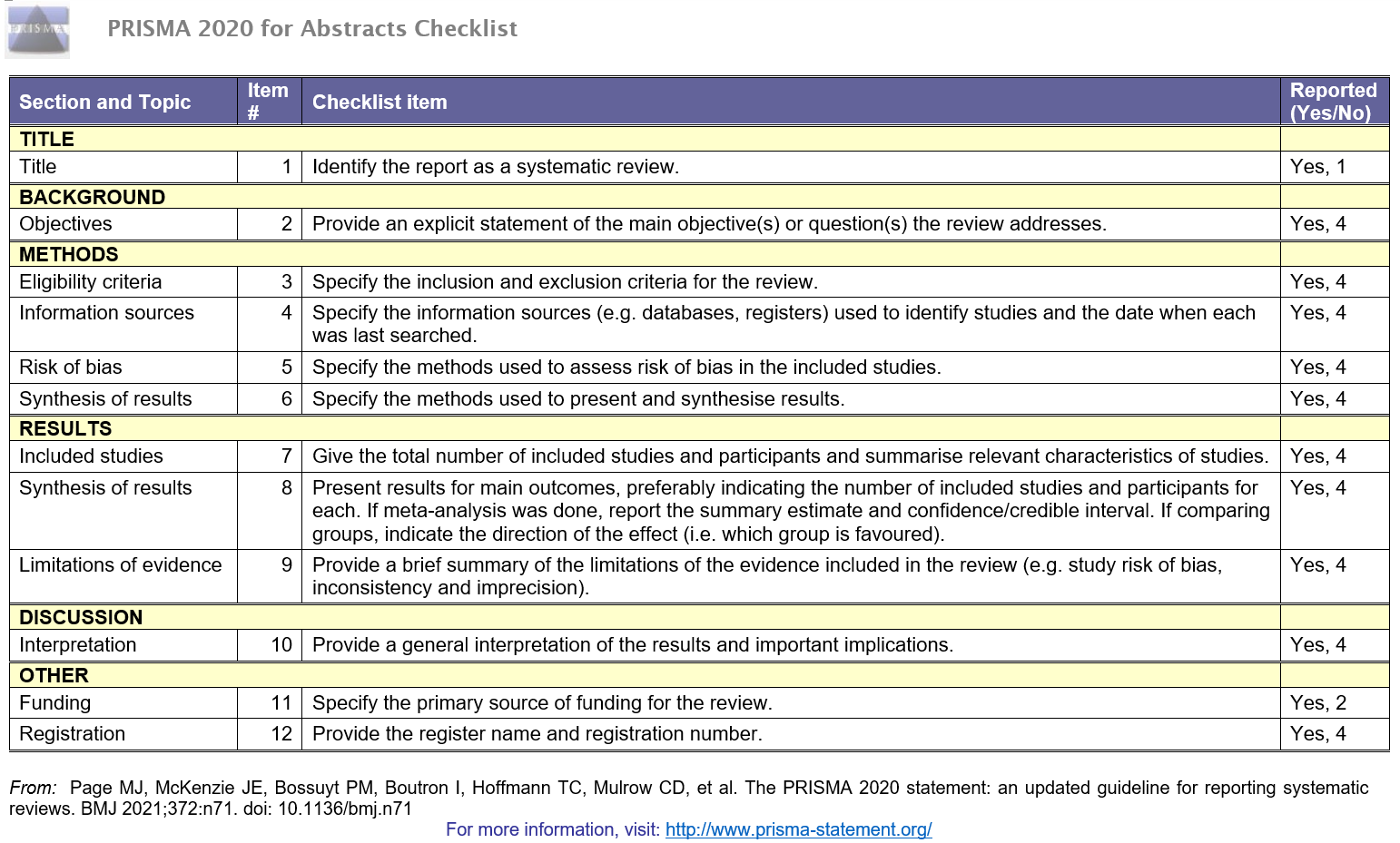


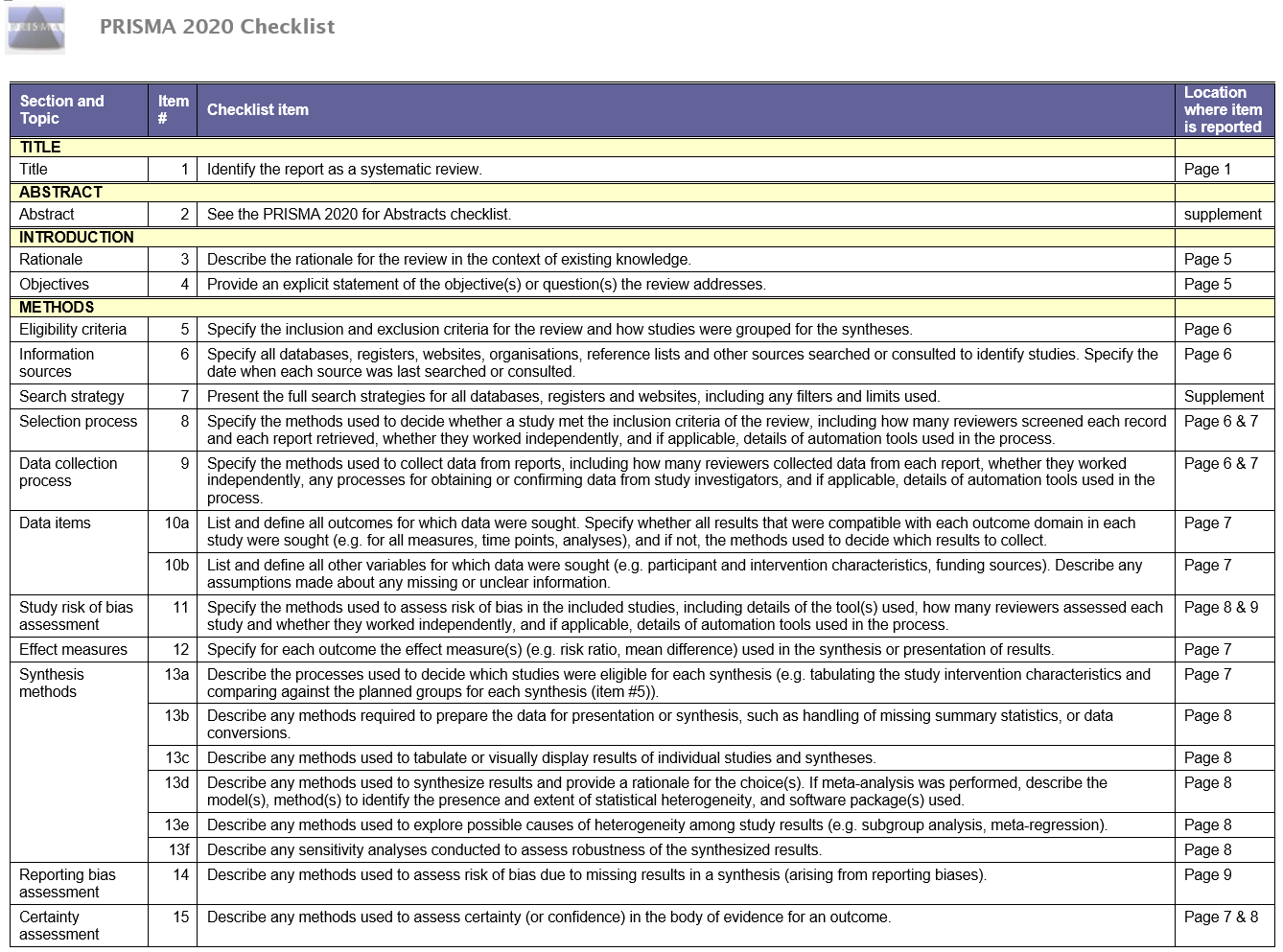


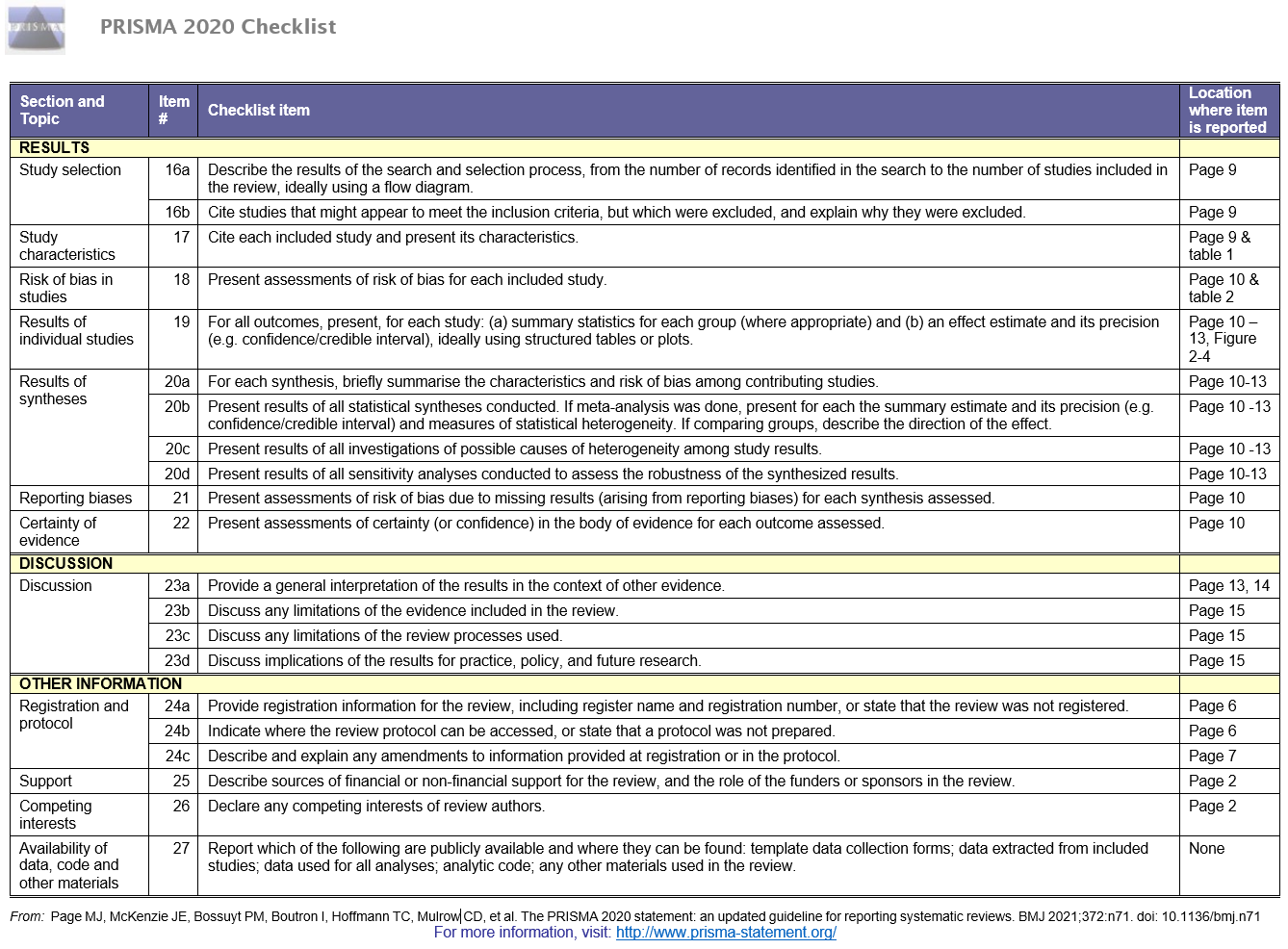

Supplement: Supplementary file 1 — Supplementary Information. [file 41598_2022_5976_MOESM1_ESM.docx]
